# Commentary: Using second-person neuroscience to elucidate the mechanisms of reciprocal social interaction

**DOI:** 10.3389/fnbeh.2020.00013

**Published:** 2020-02-11

**Authors:** Hila Z. Gvirts Probolovski

**Affiliations:** Department of Behavioral Sciences and Psychology, Ariel University, Ariel, Israel

**Keywords:** reciprocal interaction, second-person neuroscience, social neuroscience, mentalizing network, social attention system

I read with interest the recently published paper by Redcay and Schilbach (2019). In this opinion article, the authors review the emerging literature of second-person neuroscientific studies, in which participants are engaged in reciprocal social interactions. One of the aims of these authors was to uncover the neural mechanism uniquely associated with this real-time reciprocal social interaction. To this end, they compared the emerging second-person neuroscientific studies with the more traditional neuroscience approach of recording brain activity while participants observed social interactions in a third- person context (rather than an interactive participant). Interestingly, this comparison highlights the role of the neural “mentalizing network” (including the ventromedial and dorsomedial prefrontal cortex and the temporoparietal junction; TPJ) within studies employing participants engaged in reciprocal social interactions- second-person studies (Konvalinka and Roepstorff, 2012; Schilbach et al., 2013), compared to studies employing conventional psychological approaches. Importantly, the authors suggest that it may underlie the feeling of involvement with the interactive partner during reciprocal social interaction (Redcay and Schilbach, 2019).

I was pleased to see that their mentalizing network has much in common with what my colleague and I recently deemed as the “mutual social attention systems” of interacting partners—i.e., the coupling between participants' TPJ and/or prefrontal cortices during social interactions (Gvirts and Perlmutter, 2019). We provide the mechanisms and several examples throughout the article of how this inter-brain neural synchrony between the “mutual social attention systems” of interacting partners facilitates more attunement and greater allocation of attention to a specific social interaction, its participants and its goals, while tuning out other potential interactions. We concluded that face-to-face naturalistic interactions seem to increase the coupling between participants' “mutual social attention systems,” resulting in more attunement, and in greater allocation of attention and involvement in a specific social interaction, its participants and its goals (Dai et al., 2018; Fishburn et al., 2018; Davidesco et al., 2019).

Notably, although our findings and the findings by Redcay and Schilbach (2019) are differentially based on distinct literature (Redcay and Schilbach (2019) on the comparison between traditional and second-person studies, and our model on interbrain synchronization studies), they both reach a similar conclusion. Both models highlight similar brain regions as playing a crucial role in supporting face-to-face reciprocal social interactions. Thus, it can be concluded that even though more experimental results are required, we appear to be on the verge of uncovering the neural mechanisms associated with real-life face-to-face interactions.

There are other common denominators between these two frameworks. For example, Redcay and Schilbach (2019) provide an extensive review on the second-person approach's contribution to studying several key components of social interaction. Similarly, our framework also focuses on different aspects of social interaction, with a main emphasis on its link with the tendency of individuals to align their motions, emotions and cognitions, observed in various everyday situations (i.e., social alignment) (Raafat et al., 2009; Shamay-Tsoory et al., 2019). Nevertheless, though Redcay and Schilbach (2019) mention brain areas that were implicated in the reviewed literature, they do not offer an integrative neurobiological model that links the mentalization network with existing neural models. As a first step in this direction, we propose an integrative neurobiological model, linking neural synchrony in the “mutual social attention systems” with a recently suggested “social alignment model” (Shamay-Tsoory et al., 2019). Importantly, we elaborate on the potential role of the neurochemical mechanisms of Oxytocin and Dopamine in modulating the loop between these two components, thereby determining why we align our behavior with anyone specific.

Finally, both frameworks highlight the potential important theoretical and clinical implications of studying social cognition within interactive brains, especially with regards to clinical populations (Schilbach, 2016; Bilek et al., 2017). By doing so, both serve to encourage and stimulate a new line of research.

## Author Contributions

The author confirms being the sole contributor of this work and has approved it for publication.

### Conflict of Interest

The author declares that the research was conducted in the absence of any commercial or financial relationships that could be construed as a potential conflict of interest.
